# Simulation Assisted Analysis of the Intrinsic Stiffness for Short DNA Molecules Imaged with Scanning Atomic Force Microscopy

**DOI:** 10.1371/journal.pone.0142277

**Published:** 2015-11-04

**Authors:** Haowei Wang, Joshua N. Milstein

**Affiliations:** 1 Department of Optics and Optical Engineering, University of Science and Technology of China, Heifei, Anhui, China; 2 Heifi National Laboratory for Physical Sciences at the Microscale, Heifi, Anhui, China; 3 Department of Chemical and Physical Sciences, University of Toronto Mississauga, Mississauga, Ontario, Canada; 4 Department of Physics, University of Toronto, Toronto, Ontario, Canada; LAAS-CNRS, FRANCE

## Abstract

Studying the mechanical properties of short segments of dsDNA can provide insight into various biophysical phenomena, from DNA looping to the organization of nucleosomes. Scanning atomic force microscopy (AFM) is able to acquire images of single DNA molecules with near-basepair resolution. From many images, one may use equilibrium statistical mechanics to quantify the intrinsic stiffness (or persistence length) of the DNA. However, this approach is highly dependent upon both the correct microscopic polymer model and a correct image analysis of DNA contours. These complications have led to significant debate over the flexibility of dsDNA at short length scales. We first show how to extract accurate measures of DNA contour lengths by calibrating to DNA traces of simulated AFM data. After this calibration, we show that DNA adsorbed on an aminopropyl-mica surface behaves as a worm-like chain (WLC) for contour lengths as small as ~20 nm. We also show that a DNA binding protein can modify the mechanics of the DNA from that of a WLC.

## Introduction

Duplex DNA is an extended polymer and its ability to form loops and folds has important consequences for gene expression and cellular function. Central to our understanding of the conformational properties of dsDNA is the intrinsic stiffness, which characterizes the rigidity of the polymer, and is quantified by the persistence length *ξ*. The persistence length is the length scale at which a polymer changes from being stiff and rigid to soft and flexible, with the larger the persistence length, the stiffer the polymer, and vice-versa. Single-molecule measurements with optical [1,2] or magnetic tweezers [3,4] can directly measure the persistence length of dsDNA by fitting to a force-extension curve. For dsDNA with long contour lengths (*L*_*C*_ > 2–3 kbp), the worm-like chain (WLC) model provides an accurate fit for the persistence length [5], with *ξ* ≈ 50 nm being the generally accepted value at physiological salt concentrations.

Force extension measurements on small dsDNA molecules (*L*_*C*_ < 2–3 kbp) become increasingly difficult for diminishing contour lengths [6,7]. Atomic force microscopy (AFM) provides an alternative approach to measuring the mechanical properties of polymers and could, in principle, probe arbitrarily short segments of dsDNA [8,9]. In scanning mode, AFM can directly image dozens of identical molecules of DNA adsorbed onto a surface, and at sub-nanometer resolution. By pooling images of many molecules, for a given microscopic model, a persistence length can be extracted from the equilibrium statistics of contour bend angles or end-to-end distances.

The attraction of working with short DNA is that one might be able to probe sequence dependent elasticity effects, difficult to resolve with longer molecules, and which have implications for our understanding of a diverse range of phenomena from protein-mediated DNA looping [10,11] to the binding of nucleosomes along chromatin fibers [12]. However, there are three main complications that arise when trying to quantify the intrinsic stiffness of DNA with AFM. First, free DNA in solution is a three-dimensional object, but to acquire two-dimensional scans via AFM the molecule must be projected upon a planar surface. Second, this projection is highly-dependent upon the functionalization of the surface, which may also interact with and distort the molecule. And third, imaging is generally performed far from physiological conditions, often in an open air environment after drying out the sample.

Despite these complications, under appropriate conditions, it has been argued that surface adsorbed DNA can equilibrate upon a substrate and that the WLC model will still provide an accurate microscopic model of the intrinsic stiffness [9,13]. Experiments on DNA shorter than, roughly, 30 nm, however, have caused a controversy as these very short fragments tend to look much more flexible than predicted by the WLC model [14]. It has been argued that this short length scale flexibility is the result of the DNA double helix forming sharp kinks [15–19], although the matter is far from settled.

In the present manuscript, we detail a method for extracting accurate measurements of the persistence length from surface adsorbed DNA. While much effort has gone into greatly improved image processing algorithms for tracing the DNA contour [9,18], we present an alternative approach. Instead, we calibrate our contour tracing routine to mock simulations of AFM measurements on two-dimensional WLC DNA so we may accurately reproduce the simulated persistence length down to contour lengths of ~20 nm. After correcting the measured contour lengths, our analysis shows that DNA adsorbed onto an APTES modified mica surface behaves like a WLC polymer for contours as short as ~20 nm. Finally, we show that the DNA binding protein H-NS, which is thought to form rigid, extended filaments along dsDNA, can modify the behavior of the DNA from that of a WLC polymer.

## Materials and Methods

### DNA amplification

DNA fragments of 485 bp were PCR amplified with Taq polymerase from a noncoding region of the *Salmonella* laboratory strain LT2 genome. The resulting segments were purified and diluted to 10 ng/μl aliquots and stored at -20°C.

### AFM sample preparation and imaging

Mica was freshly cleaved with Scotch tape. 50 μl of 3-aminopropyl-triethoxysilane (APTES) (0.1% V/V) was dropped onto a mica surface and incubated for 10 minutes. After washing with 400 μl of milli-Q water, the mica was dried with compressed air. 50 μl of glutaraldehyde (1.0% V/V) was spread over the mica surface and incubated for 15 minutes. The mica was then washed with 400 μl of milli-Q water and air dried. Prepared mica surfaces were kept in a vacuum desiccator for up to one week.

Before imaging with the AFM, 5–10 ng of DNA was mixed with from 0–600 nM of H-NS protein in a Tris-buffer (50mM of Tris-HCl, 150 mM of KCl, pH. 7.8). The mixture was incubated at room temperature for 10 minutes to allow the protein to bind with the DNA. The solution was then deposited onto a pretreated mica surface and incubated for 20 minutes at room temperature. The sample was then rinsed with 400 μl of milli-Q water and air dried. Coated mica plates were left in a vacuum desiccator for 30 minutes to eliminate remnant moisture.

A series of 512×512 nm^2^ AFM images (FlexAFM, Nanosurf) were taken under dynamic force (tapping) mode at a 1.3 Hz scan rate with aluminum reflex coated silicon tips (BudgetSensors, Tap150Al-G). The applied force was carefully adjusted to avoid dragging the DNA by the tip while the integral gain was tuned to provide the sharpest possible images. Raw images were subsequently processed to remove the background slope, but were otherwise left unfiltered.

### Tracing and image analysis

AFM images were analyzed by a custom Matlab routine for unbiased, high-throughput analysis. Our tracing routine is based upon an algorithm originally developed by Brugal and Chassery [20] and employed by several other groups [21,22]. In brief, the image was first thresholded to create a binary map. A thinning process was then applied to generate traces of 1-D (single-pixel) contours for each DNA molecule by iteratively removing pixels along the edge of each course trace, but without breaking up the contours. Results of the automated traces were manually inspected, and bad traces were deleted or fixed by connecting or inserting breaks. In order to obtain accurate contour lengths from the pixelated traces of the DNA, the Freeman estimator [23] was used in all our analyses.

### Monte-Carlo simulations

Sets of simulated AFM images were generated using a Monte-Carlo algorithm to calibrate our experimental measurements of DNA contour length. The simulation was based on a 2D equilibrium worm-like-chain model [13]. A polymer chain with a 0.34 nm step length and 2 nm diameter was first generated using the probability distribution function for the bending angle *θ*:

$$P{\left(\theta \left(l\right)\right)}_{2D}=\sqrt{\frac{\xi }{2\pi l}}\text{exp}\left(-\frac{\xi \theta^{2}}{2l}\right),$$ (1)

where *ξ* is the persistence length and *l* is the length along the contour.

The simulated DNA was then artificially scanned by a circular tip of radius *R*_*T*_ to generate mock AFM images. While the manufacturer only stipulates that the tips are less than 10 nm in radius, a measure of the tip radius can be extracted from our actual experiment by scanning the DNA cross-section [24]; however, for a soft structure like DNA, this approach is not very accurate. Moreover, the tip size will vary from experiment to experiment. Fortunately, we found that the simulated results were relatively insensitive to our choice of tip size, which was set at *R*_*T*_ = 3 nm for the data presented. Finally, random Gaussian noise, the statistics of which are extracted from areas in our experimental AFM traces free of DNA, was applied to the simulated data to better mimic the true AFM images.

## Results and Discussion

### DNA contour length calibration

**Fig 1:**
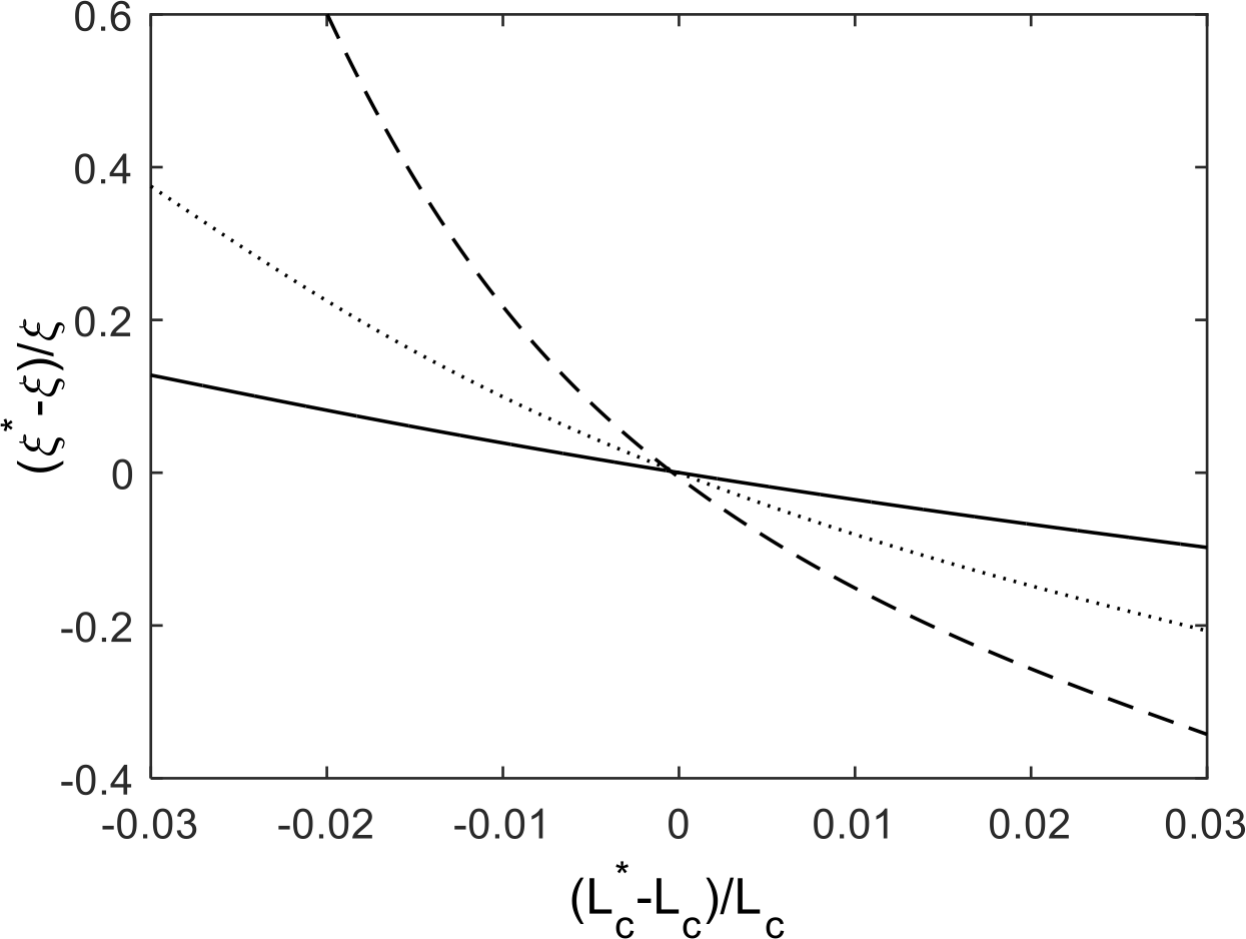
Relative error in the measured persistence length *ξ* ^*^ as a function of the relative error in the measured contour length *L* ^*^_C_. The three curves are for 100 bp (dashed), 300 bp (dotted) and 500 bp (solid) DNA. Here, *ξ* and *L*_*C*_ are the actual persistence and contour lengths, respectively.

Statistical mechanics may be used to infer mechanical properties of biological polymers like DNA. A straightforward approach is to consider the root mean square (RMS) end-to-end distance <R^2^> of an ensemble of identical polymers. Assuming the DNA to be at thermal equilibrium, the 2D worm-like chain (WLC) model provides a relationship for the mean end-to-end distance:

$$\langle R^{2}\rangle \;=4\xi L_{C}\left(1-\frac{2\xi }{L_{C}}\left(1-e^{-\frac{L_{C}}{2\xi }}\right)\right)\;,$$ (2)

where *ξ* is the persistence length and *L*_*C*_ is the contour length. This nonlinear relationship needs to be inverted for *ξ* after measuring both the RMS end-to-end distance and the contour length. Errors in contour length estimation, arising from the quality of AFM imaging and DNA tracing, increasingly contribute to uncertainty in persistence length measurements on short DNA (see Fig 1). Already, for 100 bp DNA, an underestimation of the contour length by ~2%, which is < 1 nm, leads to a ~60% overestimation of the persistence length.

In addition, these errors will greatly depend upon the stiffness of the molecule because a softer molecule will tend to curve back and forth along the surface more than a stiff molecule. Spatial details can be lost, to a certain extent, due to pixilation and blurring when imaging with AFM leading, in general, to an underestimation of the contour length. So measuring the persistence length of softer molecules requires an increasingly accurate estimate of the contour length.

To account for errors in measuring the contour length, we calibrate our measurements against simulated sets of increasingly stiff, two-dimensional WLC DNA of different lengths. We then assume that the simulated contour length *L*_*S*_ is related to the measured contour length *L* ^*^_C_ as follows:

$$L_{S}=L_{C}^{*}/f\left(\xi \right)+C,$$ (3)

where *f*(*ξ*) is a persistence length dependent function characterizing our underestimation of the actual contour length and *C* is a constant. Note, the functional form of Eq 3 was found empirically and may vary for different microscopic models or tracing algorithms. The constant arises from a contour and persistence length independent bias of our tracing algorithm originating in how we specify the end-points of each DNA molecule. Eq 3 can be solved for *C* by analysing two simulated sets of molecules with the same persistence length, but different contour lengths. If the measured contour lengths differ by a factor of α (i.e., $L_{c}^{*}=\alpha L_{C}^{\prime *}$), then the simulated contour lengths *L’*_*S*_ and *L*_*S*_ are related as $\alpha L_{S}^{\prime }-L_{S}=\left(\alpha -1\right)C$. With this approach, for our data we find *C* = 0.7428 nm.

If we let *L*_*C*_ = *L*_*S*_-*C*, to remove this constant bias, then the function *f*(*ξ*) may be defined as follows:

$$f\left(\xi \right)\equiv L_{C}^{*}/L_{C}\;.$$ (4)

**Fig 2:**
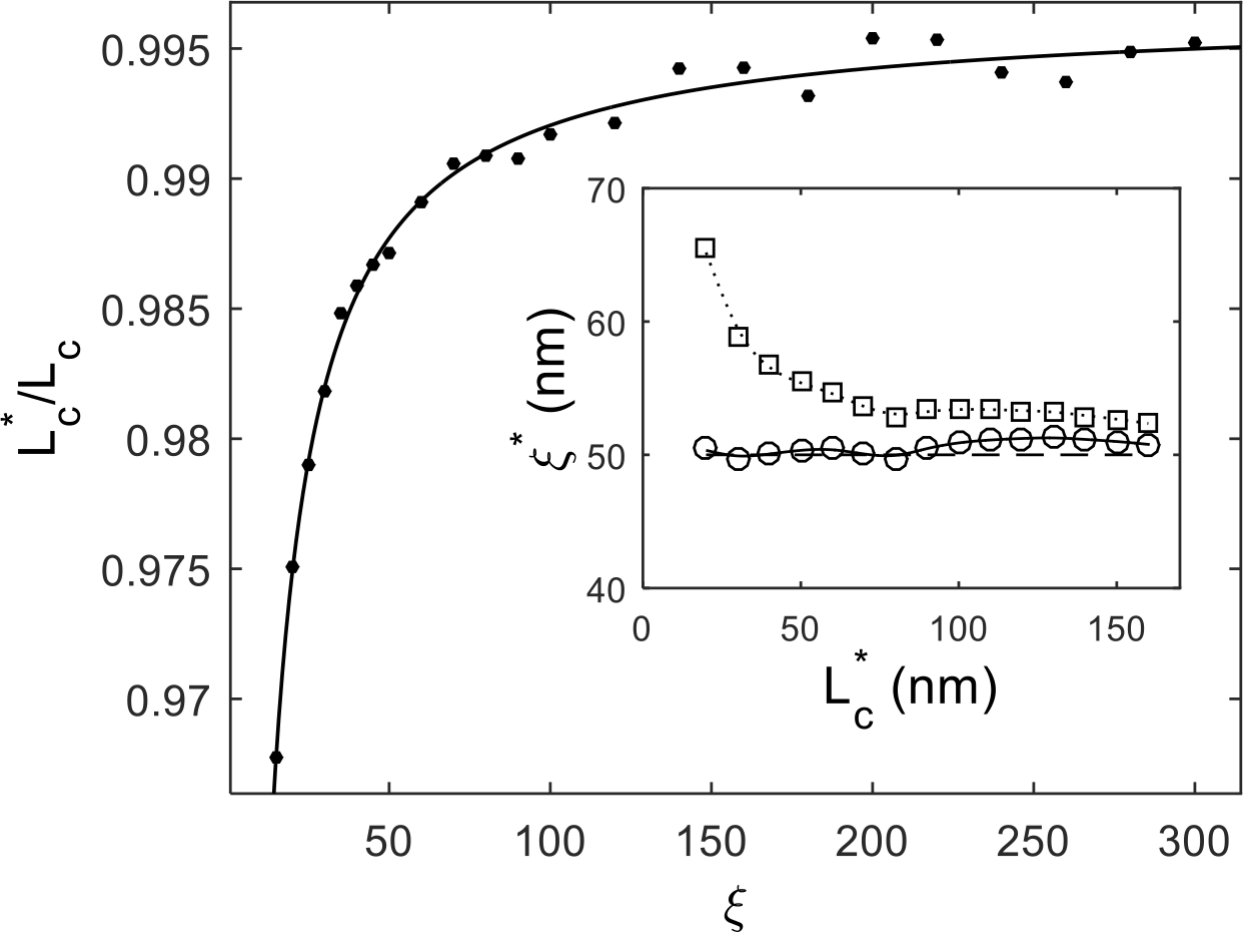
Measured contour length *L* ^*^_C_ normalized to the actual contour length *L*_*C*_ as a function of the simulated persistence length *ξ*. The solid line is a fit to the data given by *f*(*ξ*) = 1 –*A*/*ξ*. (Insert) The measured persistence length *ξ* ^*^ as a function of the measured contour length *L* ^*^_C_ for simulated 500bp/170nm DNA. The circles (squares) are data points for the calibrated (uncalibrated) contour lengths. The dashed line is at the simulated persistence length of 50 nm.

Plotting the right-hand side of Eq 4 at varying stiffness (see Fig 2) confirms that our tracing algorithm increasingly underestimates the true contour length for softer and softer DNA molecules. We find the data to be well described by the following analytical function:

$$f\left(\xi \right)=1-\frac{A}{\xi },$$ (5)

with fit parameter *A* = 0.4310 nm.

Assuming that our microscopic 2D WLC model is correct, these results can be used to correct the contour lengths we measure in our actual, physical AFM experiments. A proper scaling is performed by substituting

$$L_{C}\to L_{C}^{*}/f\left(\xi^{*}\right)\;\text{and}\;\xi \to \xi^{*}$$ (6)

into Eq 2. Again, we add an asterisk to signify that the persistence length *ξ* * is a measured value. Note, the bias term only has to be accounted for in the initial calibration. Eq 5 should now correctly scale between the actual (*L*_*C*_) and measured (*L* ^*^_C_) contour lengths in an AFM experiment. This scaling adjustment will significantly reduce the error in our persistence length measurements if the DNA does indeed behave like a 2D WLC.

### Fitting the persistence length

**Fig 3:**
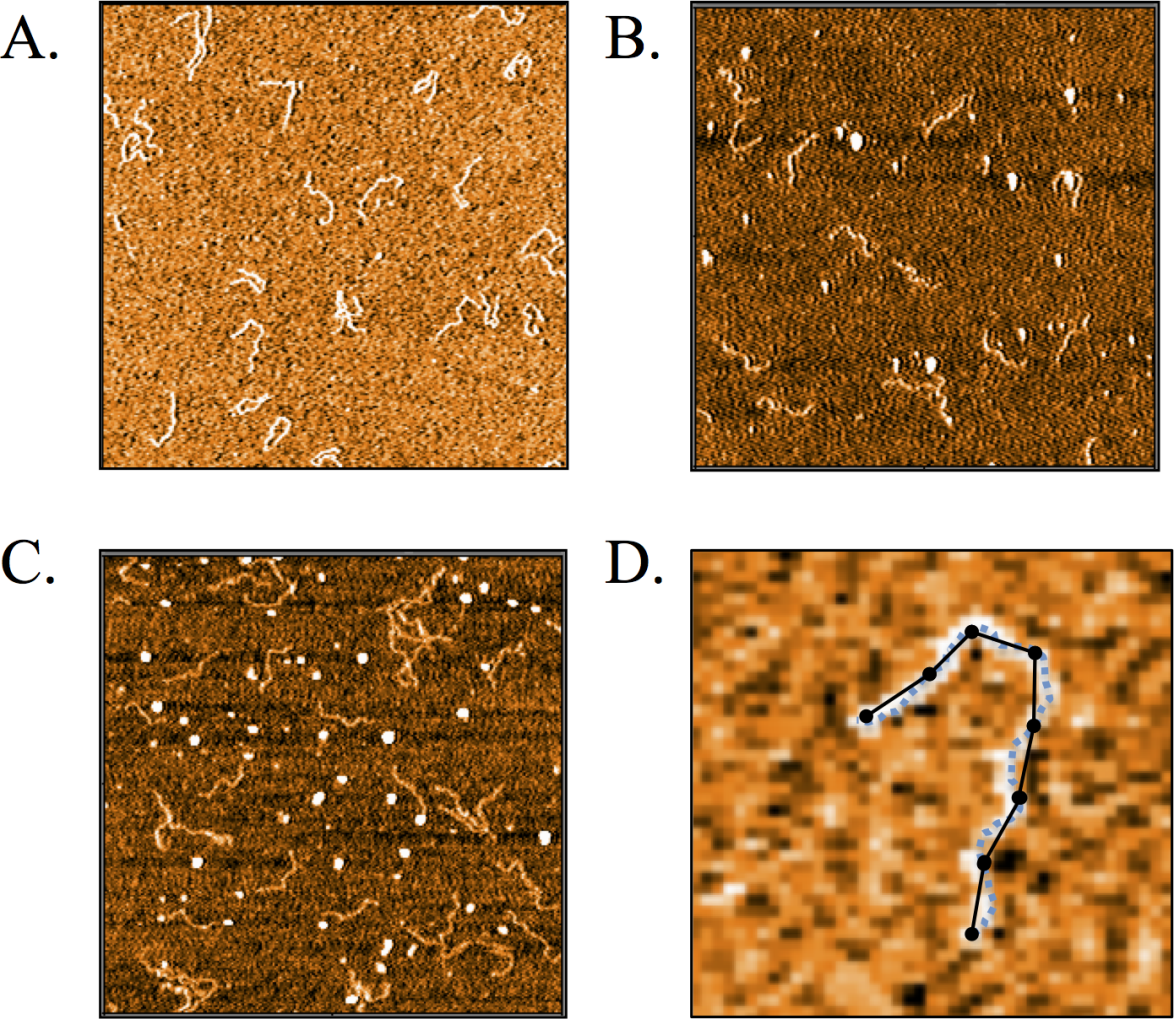
AFM images of 485 bp DNA on APTES modified mica: A.) Bare DNA, B.) DNA bound by 200 nM H-NS, and C.) 600 nM H-NS. D.) Illustration of sectioning and tracing the DNA. The dashed line traces the DNA contour and the solid lines represent the segment end-to-end distances.

Measuring the distance between endpoints of each DNA molecule within an ensemble yields one data point per molecule. However, information about the intrinsic stiffness is also contained within the internal orientations of the polymer (see Fig 3A and 3D). An analysis of the intervening DNA contour may, therefore, permit us to acquire the same precision in measuring the persistence length with a smaller sample size. Our approach is to divide the contour into neighbouring segments of equal length, which don’t overlap, and to measure the mean end-to-end distance of these segments.

In the insert to Fig 2, we plot the measured persistence length vs. segment contour length after an analysis of our mock AFM simulations with microscopic parameters *L*_*C*_ = 170 nm and *ξ* = 50 nm. The segments were determined by starting at one end of each DNA molecule and tracing the contour for a distance *L* ^*^_C_. This procedure was performed iteratively, until no additional complete segments were possible. The segments were initially pooled and fit to Eq 2. Fig 2 shows that, although the WLC was the microscopic model in our simulations, our tracings of the DNA contours introduce significant errors. After applying Eq 6 to correct the measured contour lengths, we are able to reliably estimate the persistence length of the simulated DNA to below 2% for contours as short as ~20 nm where errors would otherwise be appreciable.

**Fig 4:**
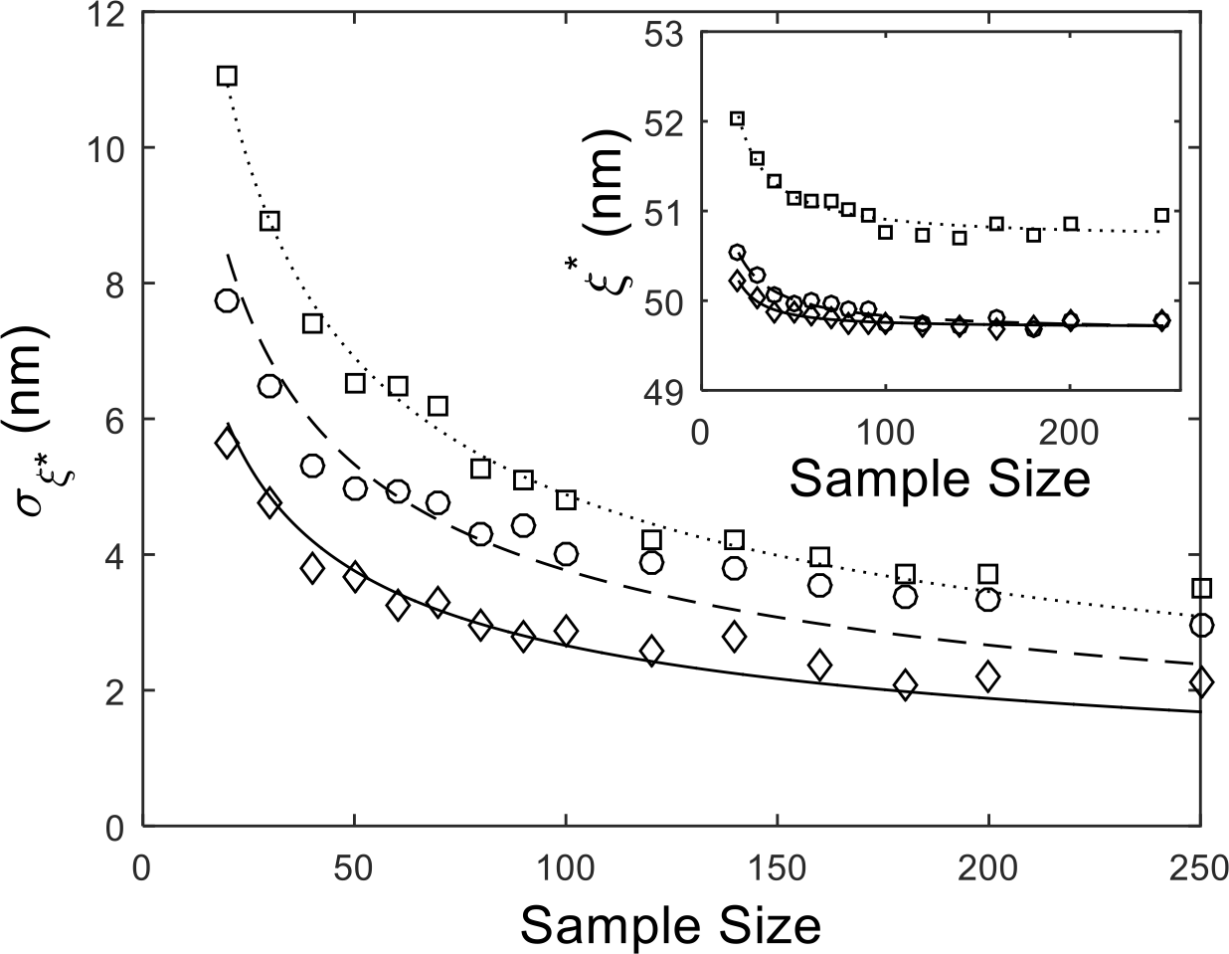
Uncertainty (standard deviation) in the measured persistence length (*σ*_ξ*_) vs. sample size for simulated DNA (*L*_*C*_ = 170 nm, *ξ* = 50 nm). The data are obtained by sectioning the DNA into segments of 30 nm (diamond), 80 nm (circle), and 160 nm (square). Solid lines are fits to *σ*_ξ*_ *~ N* ^*-1/2*^, where *N* is the sample number. The insert displays the same for the mean persistence length.

We also see that our sectioning method does indeed require a smaller sample size (i.e., number of DNA molecules) to attain the same precision. Fig 4 shows the standard deviation in the measured persistence lengths from our mock AFM simulations (again, *L*_*C*_ = 170 nm and *ξ* = 50 nm) as a function of sample size. Initially the error scales like 1/*n* ^1/2^, where *N* is the sample size, for different segment lengths. The curves are essentially scaled by a constant factor of 1/*n*_*S*_^1/2^, where *n*_*S*_ is the number of segments collected per DNA, which is a trivial result because the true sample size is the total number of DNA segments. However, systematic errors eventually cause the error to saturate for large sample number and are clearly less prevalent for shorter DNA segments. This result could be due, for instance, to various sources of drift in the sample during imaging While the increased precision is a potential benefit, we should note that the accuracy of the measurement is still determined by our earlier contour length calibration (Fig 2), which is illustrated in the insert to Fig 4.

### Intrinsic stiffness of protein bound DNA

**Fig 5:**
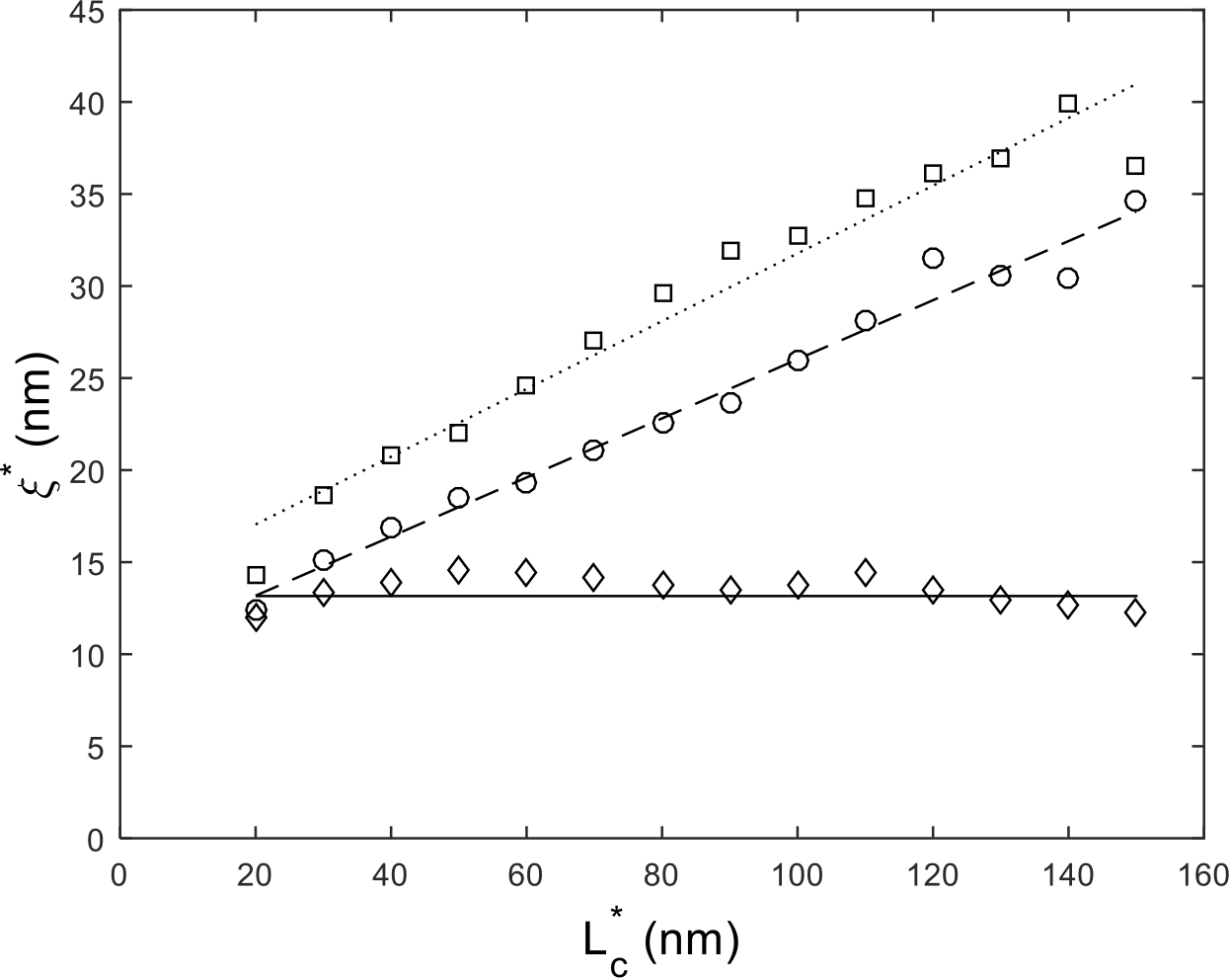
Persistence length vs. contour segment length for surface adsorbed DNA on APTES modified mica substrates. The points represent: bare DNA (diamonds), DNA bound by 200 nM H-NS (circles) and 600 nM H-NS (squares). The lines are linear fits to the data.

Finally, we apply this technique to experimental AFM measurements of 485 bp dsDNA absorbed onto an APTES coated mica surface (see Fig 3). Approximately 400 DNA molecules were imaged and traced through our automated routine. After calibrating for errors in estimating the contour length, as discussed, we plot in Fig 5 the persistence length vs. segment contour length. While the persistence length differs from the 50 nm value found in solution, we see that the WLC provides a consistent value of *ξ ≈* 13 nm at length scales as small as ~20 nm.

Adsorbing DNA onto a charged surface and then drying it should drastically effect the molecules conformational properties. It’s quite surprising then that the WLC appears to work so well. In fact, our simulation assisted procedure assumes *a priori* that the WLC is the appropriate model to describe surface adsorbed DNA. Note, our approach could easily be extended to more complex models of DNA flexibility that account for internal defects, sharp bends induced by ligands, etc. [25–27]. Nonetheless, in the current study, without applying the corrections to our experimental measurements found from the simulated, mock AFM data of WLC DNA, the experimental measure of persistence length vs. contour length presented in Fig 5 would no longer remain constant. This result lends strong support to our approach, but a better understanding of the polymer physics of surface adsorbed DNA is clearly needed.

Next we imaged DNA bound by the heat-stable nucleoid binding protein (H-NS). Found in many gram-negative bacteria, H-NS is thought to regulate gene transcription by binding to and cooperatively forming filaments along dsDNA[28]. While H-NS does not prefer a unique target sequence, like many transcription factors, it does display a greater affinity to certain sequences and has an overall preference to AT-rich DNA. Force spectroscopy with optical and magnetic tweezers has shown that the intrinsic stiffness of dsDNA is greatly increased in the presence of H-NS [29–31].

Fig 5 shows that surface adsorbed DNA bound by H-NS clearly deviates from the WLC. At 200 nM H-NS, the molecule appears increasingly stiff at long length scales, converging to the bare DNA stiffness at short length scales. At a saturation level of 600 nM, while the protein bound DNA stiffens at all length scales, the general trend persists. We should also note that free protein tended to aggregate on the mica surface, as can be seen in Fig 3A–3C, which may have altered the true protein concentration.

## Conclusions

Our simulation assisted corrections to AFM data show that inaccuracies in estimating the contour length of DNA molecules can be a major source of error in determining the intrinsic stiffness of short DNA. For air dried, surface absorbed DNA on APTES modified mica substrates, we find the molecule to behave like a WLC polymer down to length scales of ~20 nm, which is the smallest length we can reliably access. This should be contrasted with recent results of surface adsorbed DNA in solution by Mazur and Maaloum [32], who found that DNA is well described by a WLC down to ~11 nm, but with a persistence length of ~50 nm (in agreement with single-molecule pulling experiments). The surface adsorbed DNA we analyzed appeared significantly softer than their DNA imaged in solution. In fact, in an earlier paper from the same group [33], DNA fragments in solution, surface adsorbed onto mica and bound by divalent Mg^2+^ ions, were also much softer than expected at elevated salt concentrations. A two-dimensional equilibrium model, as we employ, yielded a persistence length of ~25 nm at 100 mM NaCl, while single-molecule pulling experiments obtain the commonly accepted ~50 nm persistence length at the same salt concentration. Contrasting these results with our experiments, which were performed in air on APTES modified mica, suggest that the reduced persistence length of ~13 nm we measured is primarily the result of electrostatic interactions with the charged surface. In addition, the non-aqueous environment could modify the DNA from its native B-form helical geometry, which may lead to further variation in the molecule’s intrinsic stiffness.

Our results on DNA bound by H-NS show that the polymer is increasingly flexible at short length scales. However, the approach presented in this manuscript leads to a natural extension. Instead of pooling all contour segments of a given size, one could instead pool segments across the population that correspond to the same region of the DNA. One would simply need to conjugate a marker to one end of the DNA so the molecule’s orientation may be distinguished [34,35]. This fine grained map of DNA stiffness might provide deeper insight into the transcriptional regulatory function of proteins like H-NS, much in the same way that chromatin immunoprecipitation (ChIP) [36] techniques have mapped out their binding distribution, but at finer resolution.

The current method could be improved with better sample statistics, improved AFM imaging, and a more accurate simulation (for instance, with a better profile/model of the AFM tip), making even shorter segments of DNA accessible to analysis. However, this approach should already aid in understanding how sequence dependent mechanical properties of DNA influence cell function.
